# Prognostic value of inflammatory biomarkers in aged patients with oral squamous cell carcinoma

**DOI:** 10.3389/fphar.2022.996757

**Published:** 2022-11-21

**Authors:** Takuya Yoshimura, Hajime Suzuki, Hirotaka Takayama, Shotaro Higashi, Yuka Hirano, Masahiro Tezuka, Takayuki Ishida, Kiyohide Ishihata, Marie Amitani, Haruka Amitani, Yasuhiro Nishi, Yasunori Nakamura, Yasushi Imamura, Etsuro Nozoe, Akio Inui, Norifumi Nakamura

**Affiliations:** ^1^ Department of Oral and Maxillofacial Surgery, Kagoshima University Graduate School of Medical and Dental Sciences, Kagoshima, Japan; ^2^ Department of Community-Based Medicine, Kagoshima University Graduate School of Medical and Dental Sciences, Kagoshima, Japan; ^3^ Department of Psychosomatic Internal Medicine, Kagoshima University Graduate School of Medical and Dental Sciences, Kagoshima, Japan; ^4^ Department of Oral and Maxillofacial Prosthodontics, Kagoshima University Graduate School of Medical and Dental Sciences, Kagoshima, Japan; ^5^ Department of Oral Surgery, Kagoshima Medical Center, National Hospital Organization, Kagoshima, Japan; ^6^ Department of Internal Medicine, Kagoshima Kouseiren Hospital, Kagoshima, Japan; ^7^ Pharmacological Department of Herbal Medicine, Kagoshima University Graduate School of Medical and Dental Sciences, Kagoshima, Japan

**Keywords:** sarcopenia, oral squamos cell carcinoma, inflammatory biomarkers, nutrition, frailty

## Abstract

**Background:** Better prognostic biomarkers for oral squamous cell carcinoma (OSCC) must be developed, particularly within the realm of clinically and frequently administered tests, to advise appropriate clinical therapy and follow-up. In this study, we retrospectively investigated which of the several inflammation-nutrition indicators might predict the prognosis of patients with OSCC.

**Methods:**  The preoperative neutrophil-lymphocyte ratio (NLR), lymphocyte–monocyte ratio (LMR), platelet–lymphocyte ratio (PLR), CRP-albumin ratio (CAR), Glasgow prognostic score (GPS), modified GPS (mGPS), prognostic nutritional index (PNI), controlling nutrition status (CONUT), and modified CONUT (mCONUT) were retrospectively evaluated using blood samples collected 1–5 days before surgery. To estimate the effect on the prognosis of tumor progression, the mean values of the markers between stages I/II and III/IV were used for subgroup analysis. The multivariate Cox proportional hazards model included all independent variables significantly associated with survival in the univariate analysis to determine the independent variables.

**Results:** A total of 112 patients (69 males and 43 females) with primary OSCC who underwent surgical treatment at our hospital were included. There were statistically significant differences in the mean values of monocytes, platelets, and albumin between stages I/II and III/IV. According to the multivariate Cox proportional hazards regression, a low PNI was associated with shorter overall survival (OS) and disease-free survival (DFS); women were associated with shorter DFS.

**Conclusion:** The pretreatment PNI had excellent predictive value for the 5-year OS and DFS of patients with OSCC. Future large-scale prospective studies with a high sample size are needed to verify our findings in OSCC patients.

## Introduction

Among all head and neck cancers (HNC), oral squamous cell carcinoma (OSCC) is one of the leading causes of cancer mortality (Siegel et al., 2021), while the incidence of OSCC has increased in many countries (Almangush et al., 2020). Despite advances in the treatment of OSCC and improved survival rates, prognosis remains a challenging issue. Oral and pharyngeal cancers are more likely than other cancers to cause functional disorders such as feeding and swallowing problems, depending on the site and extent of surgery, and there is also a higher risk of progression to nutritional disorders, frailty, and sarcopenia, all of which can have a negative impact on the prognosis (Pressoir et al., 2010; Swartz et al., 2016). Therefore, it is clinically important to develop better prognostic biomarkers for OSCC, especially within the scope of clinically and routinely performed tests, to guide appropriate clinical treatment and follow-up (Jiang et al., 2021).

Numerous previous studies have reported that systemic inflammation *via* host-tumor interactions and nutrition are intimately involved in cancer development and progression. Serum inflammatory-nutritional markers are widely used as a method of nutritional assessment related to the prognosis of various types of cancer and can be assessed by a combination of serum nutrient indices, inflammatory response indices, and blood cell components in routine blood examinations for cancer patients (Yamamoto et al., 2021). Inflammatory nutrient markers are a combination of high expression markers such as neutrophils, platelets, monocytes, and C-reactive protein (CRP) and low expression markers such as lymphocytes and albumin (Alb). All of these markers are measured in routine blood tests of cancer patients and are readily available (Yamamoto et al., 2021). Accumulating evidence suggests that inflammation-nutrition markers, such as neutrophil-lymphocyte ratio (NLR), lymphocyte–monocyte ratio (LMR), platelet–lymphocyte ratio (PLR), Glasgow prognostic score (GPS), CRP-albumin ratio (CAR), prognostic nutritional index (PNI), and controlling nutrition status (CONUT) are representative examples and have been reported to be associated with prognosis in various types of cancer.

Additionally, there is increasing evidence supporting the role of inflammatory-nutritional markers in prognosis associated with OSCC, especially in NLR, LMR, PLR, CAR, and PNI (Acharya et al., 2017; Chen F. et al., 2017; Lim et al., 2017; Bruixola et al., 2018; Kao et al., 2018; Bao et al., 2020; Yoshida et al., 2020; Jäwert et al., 2021; Keinänen et al., 2021; Watabe et al., 2021), while there are still few reports on their application to assess prognosis in patients with OSCC. Although the prognostic value of inflammation-nutrition markers is becoming clearer, it is still controversial which markers are useful for predicting prognosis. Therefore, in this study, we retrospectively investigated which of the multiple inflammation-nutrition markers reported thus far are relevant in predicting the prognosis of patients with OSCC.

## Materials and methods

### Patient characteristics

The study included 112 patients (69 men and 43 women) with primary OSCC who had surgical treatment at Kagoshima University Hospital between January 2009 and December 2015 were enrolled in this retrospective cohort study. The following clinicopathological characteristics were collected for all patients: sex, age, tumor site, TNM classification, tumor stage (according to the American Joint Committee on Cancer [AJCC] Cancer Staging Manual, Seventh Edition), therapy, survival rate, and duration of follow-up.

### Ethics and informed consent statement

This retrospective cohort study was carried out following the Helsinki Declaration principles and was approved by Kagoshima University’s Institutional Ethics Committee (approval No. 29–14). Due to the nature of this study, patient consent was waived. An opt-out method was applied to gain permission for this study, with a notice put on the website of Kagoshima University Hospital. The poster was accepted by Kagoshima University’s Institutional Ethics Committee.

### Serum inflammatory-nutritional markers and scores

Blood tests for CRP (mg/dl), Alb level (g/dl), neutrophil counts (cells/mm^3^), lymphocyte counts (cells/mm^3^), monocyte counts (cells/mm^3^), platelet counts (cells/mm^3^), total cholesterol (mg/dl), and hemoglobin (mg/dl) were obtained 1–5 days before surgery. There was no history of inflammation, use of the drug, or biopsy at 2 weeks which may impact the serum inflammation index. The NLR, CAR, PLR, and LMR were allocated as previously described (Chen J. H. et al., 2017; Fu et al., 2018; Zhang et al., 2019). In brief, the NLR was determined by dividing the peripheral neutrophil counts by the lymphocyte count. CAR was calculated as the ratio of CRP to Alb. The neutrophil count to lymphocyte count ratio was used to estimate the NLR. The platelet count to lymphocyte count ratio was termed PLR. The lymphocyte count to monocyte count ratio was defined as the LMR. The GPS score was calculated using a previously described method [20]. In brief, patients with low albumin concentrations (<3.5 g/dl) and high blood CRP levels (>1.0 mg/dl) were classified as GPS = 2. Those who had only one of the aberrant readings were assigned GPS = 1, whereas patients who had neither were assigned GPS = 0. GPS was modified such that patients with hypoalbuminemia were allocated a mGPS score of 0 in the absence of an elevated CRP concentration (Fan et al., 2016; Yoshimura et al., 2020). The PNI was calculated as follows: 10 × Alb (g/dl) + 0.005 × lymphocyte counts (Onodera et al., 1984). The Alb, total cholesterol, and lymphocyte counts were used to determine the CONUT scores (Kono et al., 2017; Jeon et al., 2020). The modified CONUT was calculated from the Alb, lymphocyte counts, and hemoglobin instead of total cholesterol. Both the CONUT score and mCONUT score were divided into normal nutrition and light (score = 0), moderate (score = 1), and severe malnutrition groups (score = 2).

### Statistical analysis

The difference was statistically significant at *p* < 0.05. Student’s t test was conducted to compare the mean values between stages I/II and III/IV for subgroup analysis. All independent variables that were significantly (*p* < 0.1) correlated with survival in the univariate analysis were included in the multivariate Cox proportional hazard model. Using receiver operating characteristic (ROC) curves, we calculated different cutoff lines for the statistically significant factors and selected the optimal cutoff values for each of them to classify the poor prognostic group. The Youden index was used to determine the best cutoff values for variable estimation. The survival curves were assessed using the Kaplan-Meier method. The log-rank test was used to compare the survival curves between the two groups. To examine the value’s predictive accuracy, a time-dependent ROC curve analysis was carried out. All analyses were conducted using Stata version 16 (StataCorp LLC, College Station, TX) and GraphPad Prism version 9.4.0 for macOS (GraphPad Software, San Diego, CA).

## Results

### Study characteristics

Table 1 summarizes the patient characteristics. Males accounted for more than half of the patients (58 percent). The patients’ average age was 68 years. Primary tumors invaded the oral mucosa in the following locations: tongue, gingiva, oral floor, buccal, palate, and lip. At the time of diagnosis, 51% of patients had advanced disease (TNM stages III and IV). Sixty-six patients were treated only with surgery, whereas 46 were treated with surgery plus concomitant chemoradiotherapy. The average duration of follow-up was 1,335 days.

**TABLE 1:** Patient characteristics.

Characteristics	n = 112
Sex (male/female)	69/43
Age (years)	68 [59–77]
Tumor location	—
Tongue/gingival/oral floor/buccal/palate/lip	52/34/11/8/6/1
Classification	—
T1/T2/T3/T4	25/59/15/13
N0/N1/N2	69/19/24
Stage	—
Ⅰ/Ⅱ/Ⅲ/Ⅳ	21/36/21/34
Treatment	—
Surgery only/Surgery with RT/CT	66/46
Disease-specific survival (%)	88.1
Disease-free survival (%)	69.9
Overall survival (%)	79.7
Follow up duration	1335 [850–1825]

Continuous data are presented as the medians (interquartile range [IQR]). RT, radiation therapy; CT, chemotherapy.

### Mean comparison between the progression of the tumor stage

**FIGURE 1:**
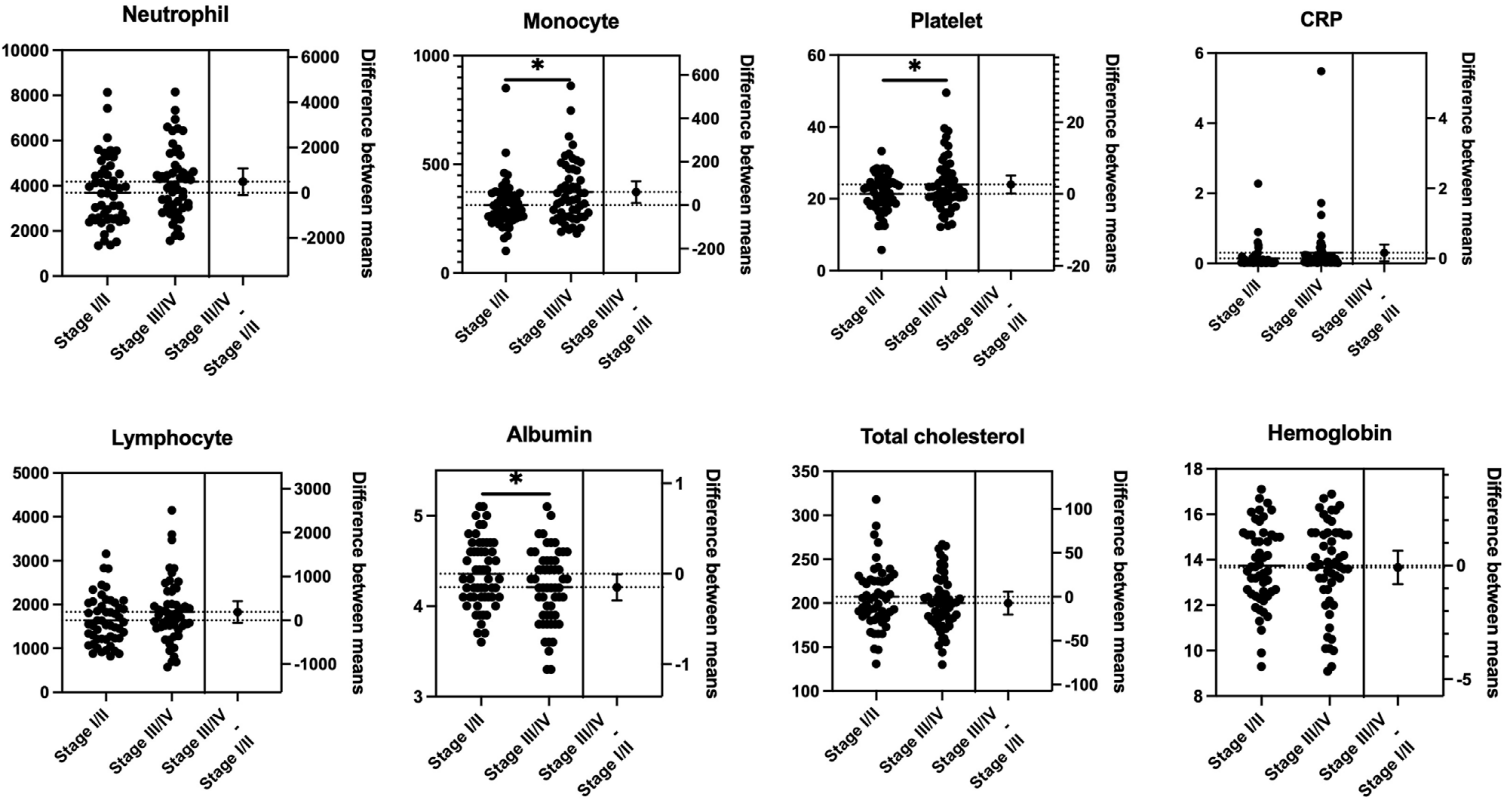
Mean comparison of blood tests between the progression of the tumor stage. Statistically significant differences were observed in the mean values of monocytes (*p* = 0.018), platelets (*p* = 0.038), and albumin (*p* = 0.045) between stages I/II and III/IV (Student’s t test).

**FIGURE 2:**
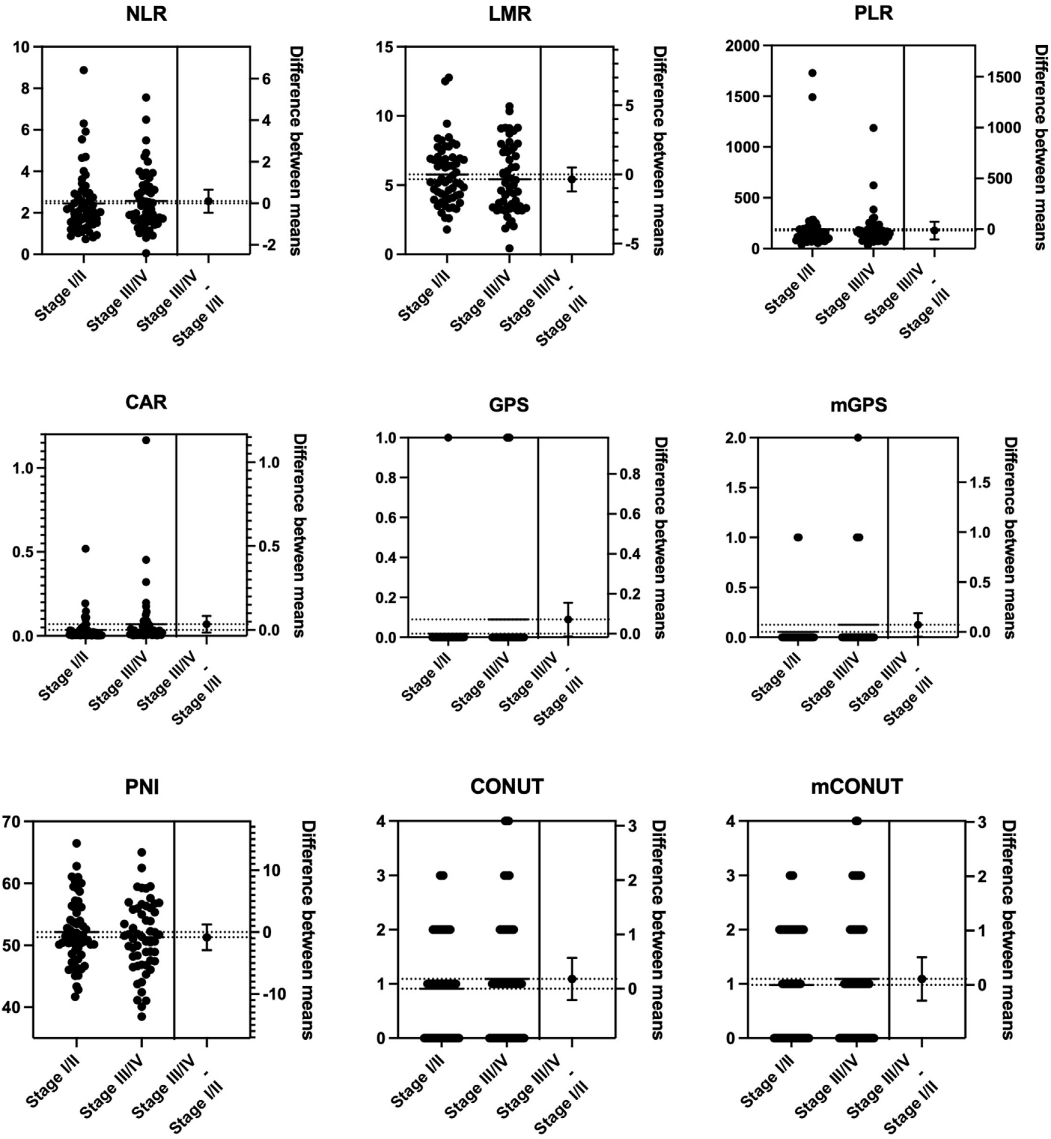
Mean comparison of inflammation-nutrition markers between the progression of the tumor stage. No significant differences were observed between stages I/II and III/IV in NLR, LMR, PLR, CAR, GPS/mGPS, PNI, or CONUT/mCONUT. PNI, prognostic nutritional index; NLR, neutrophil-lymphocyte ratio, LMR, lymphocyte–monocyte ratio; PLR, platelet–lymphocyte ratio; CAR, CRP-albumin ratio; GPS, Glasgow prognostic score; mGPS, modified Glasgow prognostic score; CONUT, controlling nutrition status; mCONUT, modified controlling nutrition status.

To estimate the effect of tumor progression, we conducted a comparison of the blood markers between stages I/II and III/IV. As shown in Figure 1, there were statistically significant differences in the mean values of monocytes (*p* = 0.018), platelets (*p* = 0.038), and albumin (*p* = 0.045) between stages I/II and III/IV. No significant differences were observed between stages I/II and III/IV in NLR, LMR, PLR, CAR, GPS/mGPS, PNI, or CONUT/mCONUT (Figure 2).

### Prognostic indicators for OS, DFS, and DSS in patients with OSCC according to a cox proportional hazards regression model for survival rates

**FIGURE 3:**
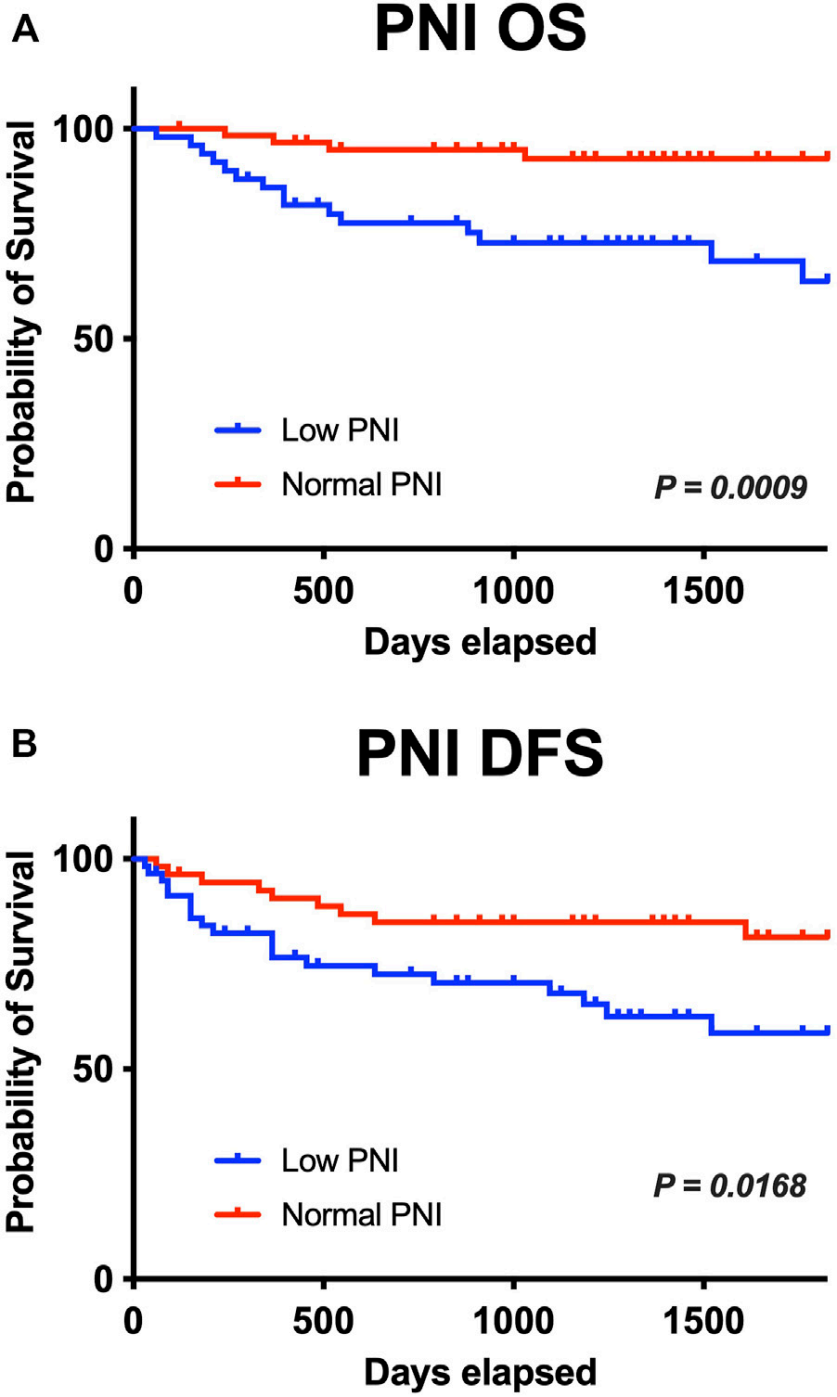
The low preoperative PNI group had lower survival rates. **(A, B)** The overall survival rate and disease-free survival rate in patients with a low PNI were significantly lower than those in patients with a normal PNI (*p* = 0.0009 and *p* = 0.0168). PNI, prognostic nutritional index; OS, overall survival; DFS, disease-free survival.

**FIGURE 4:**
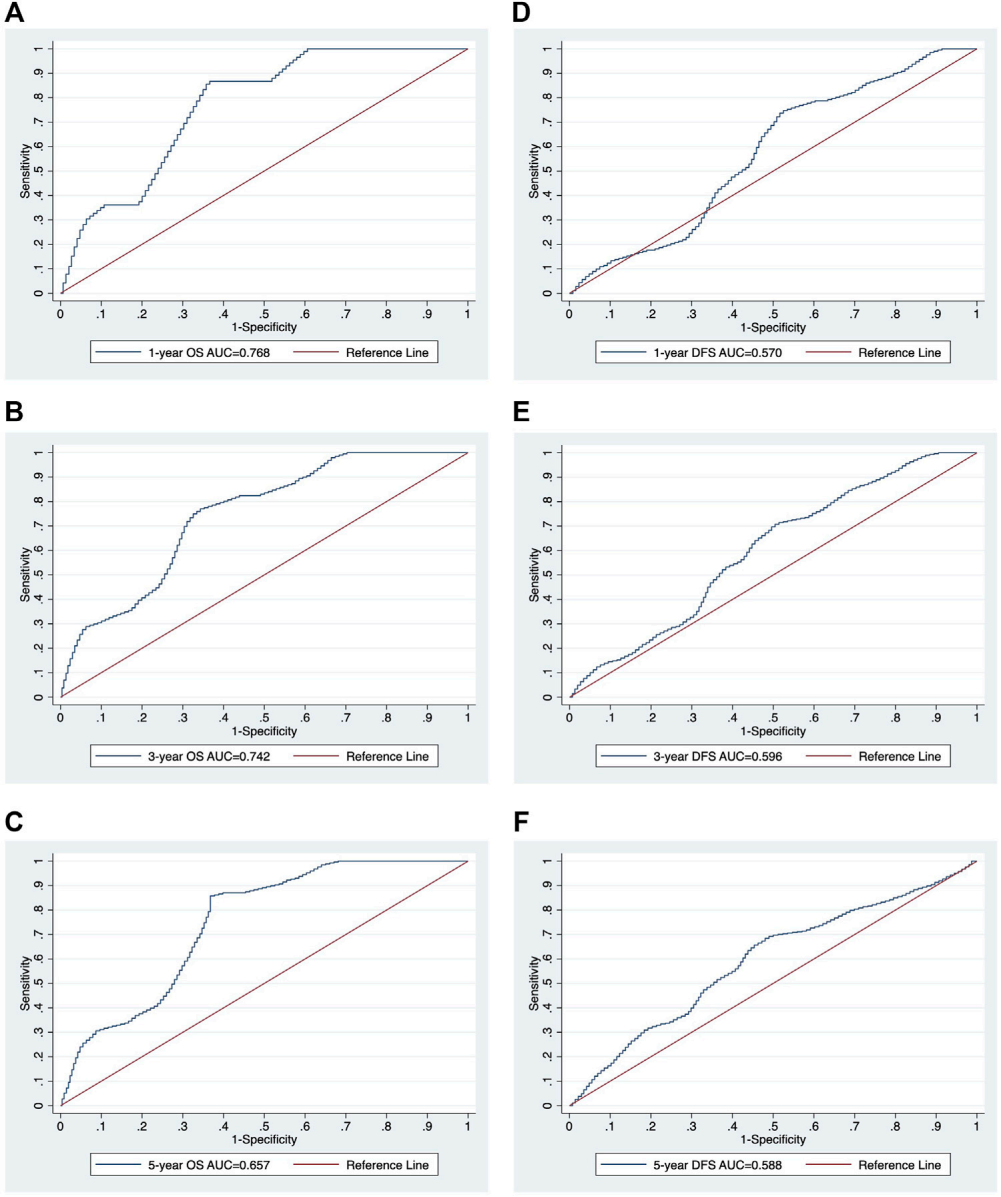
The time-dependent ROC curve analysis of PNI for 1-, 3-, and 5-year survival. The ROC curves of PNI for 1-, 3-, and 5-year survival OS **(A-C)** and DFS **(D-F)**. ROC, receiver operating characteristic; AUC, area under the curve; PNI, prognostic nutritional index; OS, overall survival; DFS, disease-free survival.

In the univariate analysis, the following were risk factors for OS: age (*p* = 0.007), PNI (*p* = 0.000), CONUT (*p* = 0.030), and mCONUT (*p* = 0.032). The risk factors for DFS were sex (*p* = 0.045) and PNI (*p* = 0.095). Additionally, the following covariates were associated with a statistically significant difference in DSS: age (*p* = 0.083) and PNI (*p* = 0.036). According to the multivariate Cox proportional hazards regression, a low PNI was associated with shorter OS (HR, 0.859; 95% CI 0.751–0.982; *p* = 0.027) and DFS (HR, 0.892; 95% CI 0.797–0.997; *p* = 0.045); women were associated with shorter DFS (HR, 1.133; 95% CI 0.355–3.621; *p* = 0.032). The corresponding HRs, CIs, and *p* values are summarized in Table 2. We calculated cutoff values of PNI for OS and DFS by ROC curves and selected the optimal cutoff values for each of them. The cutoff values of PNI for OS and DFS were 50.61 (area under the curve (AUC) = 0.7538; sensitivity, 84.21%; specificity, 62.37%) and 51.52 (AUC = 0.5866; sensitivity, 68.97%; specificity, 54.22%), respectively. Patients were divided into two groups based on their PNI levels: “low PNI,” which was below the cutoff values, and “normal PNI,” which was above the cutoff values. The survival rates in patients with a low PNI were significantly lower than those in patients with a normal PNI in both OS and DFS (*p* = 0.0009, *p* = 0.0168; Figure 3). The time-dependent ROC curves of PNI for predicting 1-, 3-, and 5- year OS and DFS are presented in Figure 4.

**TABLE 2:** Univariate and multivariate Cox proportional hazards assessments for prognosis in patients with OSCC.

Variable	OS				DFS				DSS			
	Univariate	Multivariate			Univariate	Multivariate			Univariate	Multivariate		
	*p*	HR	95%CI	*p*	*p*	HR	95%CI	*p*	*p*	HR	95%CI	*p*
Age	0.007	1.052	0.999–1.107	0.051	0.456	—	—	—	0.083	1.005	0.974–1.037	0.728
Sex (Female = 1)	0.493	—	—	—	0.045	1.133	0.355–3.621	0.032	0.484	—	—	—
PNI	0.000	0.859	0.751–0.982	0.027	0.095	0.892	0.797–0.997	0.045	0.036	0.946	0.882–1.015	0.125
NLR	0.563	—	—	—	0.541	—	—	—	0.609	—	—	—
LMR	0.315	—	—	—	0.462	—	—	—	0.428	—	—	—
PLR	0.504	—	—	—	0.889	—	—	—	0.461	—	—	—
CAR	0.983	—	—	—	0.435	—	—	—	0.902	—	—	—
GPS	0.161	—	—	—	0.798	—	—	—	0.452	—	—	—
mGPS	0.224	—	—	—	0.740	—	—	—	0.309	—	—	—
CONUT	0.030	1.376	0.674–2.809	0.380	0.401	—	—	—	0.472	—	—	—
mCONUT	0.032	0.689	0.346–1.372	0.290	0.575	—	—	—	0.483	—	—	—

PNI, prognostic nutritional index; NLR, neutrophil-lymphocyte ratio; LMR, lymphocyte–monocyte ratio; PLR, platelet–lymphocyte ratio; CAR, CRP-albumin ratio; GPS, glasgow prognostic score; mGPS, modified Glasgow prognostic score; CONUT, controlling nutrition status; mCONUT, modified controlling nutrition status; HR, hazard ratio; CI, confidence interval.

## Discussion

In this study, we retrospectively examined inflammation-related markers and nutritional markers from blood samples before primary surgery regarding the prognosis of patients with OSCC. We found that the more advanced the stage was, the worse the monocyte, platelet, and albumin levels. We also found that PNI showed prognostic value in 5-year overall survival and disease-free survival for patients with OSCC.

Although the TNM staging system is well established as a predictive clinical parameter in terms of guiding therapy and clinical prognosis for most head and neck cancers, including OSCC, it only considers tumor-related characteristics to stratify patients into prognostic groups (Valero et al., 2020b). Based on several observational studies, clinical parameters, such as TNM staging, a subsite of the tumor, sex, age, and smoking status, have limited predictive utility for head and neck cancers (Li et al., 2013). Therefore, pretreatment prognostic indicators of treatment efficacy and subsequent survival are becoming increasingly essential (Ye et al., 2020). Numerous previous studies have reported that the cancer-associated systemic inflammatory response and nutritional status are critical indicators of tumor progression and effectively predict the prognosis of various types of cancer (Jiang et al., 2021). Nevertheless, it is still unknown whether a single marker has the most predictive value or whether a combination of numerous markers performs better in terms of OSCC prognosis (Ye et al., 2020).

Inflammation plays an essential role in the development and progression of malignant tumors. Furthermore, malnutrition has been linked to carcinogenesis, cancer development, tumor progression, and tumor prognosis (Jiang et al., 2021). Several proinflammatory cytokines are produced by both the tumor cell and host stroma, which recruit inflammatory cells such as neutrophils and monocytes. In several cancers, including head and neck cancers, increased peripheral neutrophil and monocyte counts have been associated with poorer prognosis (Valero et al., 2017). Valero et al. also reported that pretreatment with peripheral blood leukocytes, particularly neutrophils, was the most robust independent predictor marker for OS in OSCC patients (Valero et al., 2020a). Cancer cells, on the other hand, can stimulate effective antitumor immunity, with T lymphocytes playing a critical role in the host immune response to cancer, and higher numbers of these lymphocytes have been linked to improved outcomes in head and neck cancer (Boscolo-Rizzo et al., 2021). Consequently, most inflammation-related markers were a combination of upregulated factors (neutrophils, platelets, monocytes, and CRP) and downregulated factors (lymphocytes, albumin, total cholesterol, and hemoglobin) in peripheral blood analysis, as summarized in Table 3. In our study, only PNI, as a surrogate marker composed of downregulated factors, was statistically significant in OS and DFS in the multivariate analysis, while no surrogate markers composed of upregulated factors were significantly correlated with prognosis. The CONUT/mCONUT scores, which were calculated by adding total cholesterol or hemoglobin to the albumin and lymphocyte counts that comprise PNI, were only statistically significant in univariate analysis. The underlying mechanism remains unclear; however, one of the reasons appears to be related to the weak differences in the mean of the upregulated factors and downregulated factors according to the tumor stages. In the subgroup analysis, only monocytes and platelets were significantly higher in stage III/IV than in stage I/II, while neutrophils, which are representative upregulated factors, were not. Neutrophils play an essential role in cancer progression, including promoting angiogenesis, immunosuppression, and cancer metastasis by secreting cytokines and chemokines and releasing reactive oxygen species (Boscolo-Rizzo et al., 2021). However, reports on the quantification of neutrophils at other HNC subsites are lacking to date (Wondergem et al., 2020). Furthermore, although the correlation between leukocytes and clinicopathologic features may have prognostic value, it is difficult to establish a consistent cutoff value for leukocytes because it depends on the clinicopathologic features of the target group. (Valero et al., 2020a).

**TABLE 3:** Representative inflammation-related markers.

	PNI	NLR	LMR	PLR	CAR	GPS/mGPS	CONUT	mCONUT
Upregulation								
Neutrophil	—	○	—	—	—	—	—	—
Platelet	—	—	—	○	—	—	—	—
Monocyte	—	—	○	—	—	—	—	—
CRP	—	—	—	—	○	○	—	—
Downregulation								
Lymphocyte	○	○	○	○	—	—	○	○
Albumin	○	—	—	—	○	○	○	○
Total cholesterol	—	—	—	—	—	—	○	—
Hemoglobin	—	—	—	—	—	—	—	○

PNI, prognostic nutritional index; NLR, neutrophil-lymphocyte ratio; LMR, lymphocyte–monocyte ratio; PLR, platelet–lymphocyte ratio; CAR, CRP-albumin ratio; GPS, glasgow prognostic score; mGPS, modified Glasgow prognostic score; CONUT, controlling nutrition status; mCONUT, modified controlling nutrition status.

It is well known that immunocompetence is decreased in hypo-nutritional and hypercatabolic states due to the reduced production of immunocompetent cells. Immunocompetence and nutritional status are closely related, and nutritional assessment methods are based on serum nutritional indices and a combination of blood cell components. Similar to previous reports, we found that a low PNI was a candidate surrogate marker in terms of poor prognosis in patients with OSCC. Low PNI is associated with poor prognosis in various studies because inflammatory cytokines such as IL-6 and IL-8 increase the number of neutrophils and decrease lymphocytes in addition to enhancing proteolysis (Abe et al., 2021), which leads to muscle wasting. Recently, we focused on nutritional status and sarcopenia in patients with OSCC and found that the combination of low PNI and the presence of sarcopenia determined by computed tomography scan was an independent predictor for DSS (Yoshimura et al., 2020). Contrary to PNI, there have been few reports that represent the efficiency of the CONUT/mCONUT score in patients with OSCC to date (Kono et al., 2017); accordingly, the efficacy of the CONUT/mCONUT score needs to be elucidated in a future study to demonstrate its prognostic significance.

To the best of our knowledge, this is the first study comparing multiple inflammation-related markers in terms of prognosis in patients with OSCC. However, there are some limitations raised in this study. First, because of this study’s limited sample size and retrospective nature, selection bias could not be completely excluded. Moreover, values were evaluated before treatment only. We did not compare the association of noncancer-related conditions with host factors. Other factors that may affect inflammatory markers, such as infections or autoimmune diseases, were not eliminated.

## Conclusion

The pretreatment PNI demonstrated excellent prognostic value in 5-year OS and DFS for patients with OSCC. Future large-scale prospective studies with a large sample size are needed to validate our findings in OSCC patients.

## Data Availability

The original contributions presented in the study are included in the article/supplementary material, further inquiries can be directed to the corresponding author.
